# The role of vitamin D in diabetic foot ulcer; an umbrella review of meta-analyses

**DOI:** 10.3389/fnut.2024.1454779

**Published:** 2024-10-09

**Authors:** Lu Liu, Fan Zhang, Mehrdad Jamali, Nathalia Sernizon Guimarães, Nima Radkhah, Parmida Jamilian, Qian Wang

**Affiliations:** ^1^Department of Orthopedics, Honghui Hospital, Xi’an Jiaotong University, Xi’an, China; ^2^Department of Orthopedics, Sun Yat-Sen Memorial Hospital of Sun Yat-Sen University, Guangzhou, China; ^3^Student Research Committee, Tabriz University Medical Sciences, Tabriz, Iran; ^4^Faculty of Nutrition and Food Science, Tabriz University of Medical Sciences, Tabriz, Iran; ^5^Department of Nutrition, Nursing School, Federal University of Minas Gerais, Belo Horizonte, Brazil; ^6^School of Pharmacy and Bioengineering, Keele University, Keele, United Kingdom

**Keywords:** serum vitamin D, diabetes, diabetic foot ulcer, meta-analyses, umbrella review

## Abstract

**Background:**

Complications related to diabetic foot ulcers (DFU) due to diabetes are increasing. One of the factors influencing the management and treatment of complications related to DFU is the vitamin D serum levels of patients. Therefore, we sought to comprehensively review meta-analyses from randomized controlled trials and observational studies examining the link between serum vitamin D levels and DFU outcomes in diabetic patients.

**Methods:**

We searched PubMed, Scopus, and ISI Web of Science until September 2024 and extracted the required data from related articles according to Inclusion criteria. The certainty of the evidence and the quality of conduct of the published meta-analyses were rated using the ASMTAR 2 tools, respectively.

**Result:**

A total of 8 meta-analyses studies that met inclusion criteria were included. Based on the obtained results, it has been noted that individuals with DFU exhibit serum vitamin D levels significantly lower, ranging from −7.14 (5.44, 8.83) to −0.93 (95% CI: 0.17, 1.68) ng/ml, compared to those with diabetes but without DFU. Furthermore, individuals exhibiting severe vitamin D deficiency are found to be at least 1.82 times more susceptible to developing DFU. Conversely, administering varying doses of vitamin D supplementation has been shown to positively affect the size and number of ulcers in DFU patients.

**Conclusion:**

This study suggests a potential link between lower levels of vitamin D in the blood and the risk of DFU, hinting at the benefits of vitamin D supplementation in improving outcomes associated with DFU. However, caution is warranted due to the potential bias present in the included studies.

## Graphical abstract

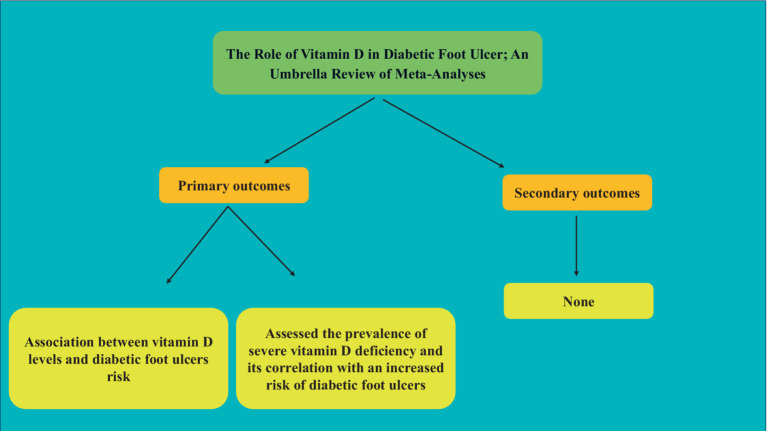

GRAPHICAL ABSTRACT

## 1 Introduction

Diabetic foot disease (DFD) represents one of the most debilitating and frequent complications among individuals with diabetes mellitus (1). The International Working Group on the Diabetic Foot defines DFD as infection, ulceration or destruction of tissues of the foot associated with neuropathy and/or peripheral artery disease in the lower extremity of a person with diabetes mellitus or clinical history (2). According to the International Diabetes Federation there are approximately 540 million adults with diabetes worldwide. This number is expected to grow to 783 million by 2045, an increase of 46% (3). A person with diabetes has a 34% lifetime risk of developing a DFU (4). The prevalence of this pathology has increased significantly in recent decades, reflecting not only the increase in the global incidence of diabetes, but also factors such as population ageing, lifestyle changes and urbanization. Waibel and colleagues (5) highlighted that the ongoing rise in diabetes prevalence is anticipated to lead to an increased demand for resources for diabetic foot disorders, encompassing both caregiver support and economic investment. DFD is associated with serious complications such as amputation and systemic infections imposing a significant burden on public health and healthcare systems worldwide (6). Diabetic foot ulcers (DFU) may result in significant emotional, physical, and financial hardship, adversely affecting patients’ and their caregivers’ quality of life. Without appropriate management, DFU, along with ischemia and infection, can lead to gangrene, lower limb amputation, and potentially death (7).

In this context, nutritional interventions have been explored as a potentially effective approach to the treatment of diabetic foot ulcers. A recent study published in the Cochrane Database of Systematic Reviews by Moore and collaborators (8) investigated the role of nutritional interventions in the treatment of diabetic foot ulcers. This systematic review examined a variety of interventions, including supplementation of specific nutrients, special diets and nutritional management strategies, with the aim of assessing their effectiveness in promoting ulcer healing and preventing further complications. While the vitamin D component of this study comprises a Randomized Controlled Trial (RCT) rather than a meta-analysis, it yields intriguing and compelling findings. The results of this study did not show a significant effect of high doses of vitamin D (in both intervention and control groups) on the improvement of DFU-related outcomes. But in low doses and compared to the placebo group, it suggested a positive effect on improving the size of the wound. In addition, recent papers explored the possible association between vitamin D deficiency and DFD. Vitamin D, a fat-soluble nutrient, is vital for regulating calcium and phosphate metabolism, crucial for maintaining strong bones and healthy teeth (9). Chronic inflammation is a significant factor in diabetes and its complications. Vitamin D has anti-inflammatory and immune-modulating properties that may help reduce inflammation and modulate the immune response, potentially lowering the risk of diabetic complications (10). Diabetic foot ulcers are a common and serious complication of diabetes, often leading to lower limb amputations (11). Vitamin D’s role in wound healing has garnered attention due to its involvement in the modulation of inflammation and infection control. Adequate levels of vitamin D may support the healing process of diabetic foot ulcers by enhancing the body’s response to infection and promoting tissue repair (12, 13). In view of these recent advances in research, it is imperative to carry out a comprehensive and critical review of the literature to assess the current state of knowledge on the role of vitamin D in the treatment and management of diabetic foot disease (12, 14).

Various meta-analyses have been performed in this field. The results of these papers show a high prevalence in relation to vitamin D levels and its relationship with the chance of DFU occurrence. Also, the quality of most meta-analyses has not been good, and considering the types of studies included in some meta-analyses, their results may not be reliable. Therefore, for a complete and comprehensive review with the aim of obtaining more accurate and reliable results and pooling the results this umbrella review aims to consolidate the available evidence on the association between level of serum vitamin D and diabetic foot ulcer. It is hoped that this work will contribute to the understanding and more effective treatment of this devastating complication of diabetes, thus improving the quality of life of affected patients.

## 2 Methods

The study followed the Preferred Reporting Items for Systematic Reviews and Meta-analyses (PRISMA) reporting guideline (15).

### 2.1 Search strategy

For relevant literature published up to September 2024, we searched electronic databases including, Scopus, Web of Science, PubMed, and Google scholar. The strategy and terms used to search for articles are as follows: (((“Vitamin D”[Mesh] OR “Ergocalciferols”[Mesh] OR “Vitamin D Deficiency”[Mesh] OR “Cholecalciferol”[Mesh]) OR ((((Vitamin D[Title/Abstract]) OR (Ergocalciferols[Title/Abstract])) OR (Cholecalciferol[Title/Abstract])) OR (“25-hydroxycholecalciferol”[Title/Abstract]))) AND ((“Diabetic Foot”[Mesh]) OR (((“diabetic foot”[Title/Abstract]) OR (“diabetic foot ulcer”[Title/Abstract])) OR (“foot ulcer” “[Title/Abstract])))) AND((meta-analysis[Publication Type]) OR (meta-analysis[Title/Abstract])). The inclusion of reviews was limited to those that were conducted in English.

### 2.2 Inclusion and exclusion criteria

The PICO criteria for the present umbrella review of meta-analyses were structured as follows: **Population/patients (P):** meta-analyses that included eligible individuals were adults aged 18 years or older with DFU, and controls without DFU; **Intervention/exposure (I):** focused on serum vitamin D levels, including interventions aimed at enhancing serum vitamin D levels, exposure to specific serum vitamin D levels, or the status of DFU; **Comparison (C):** control group or placebo; **Outcome (O):** DFU-related outcomes included pertinent factors such as DFU healing (percentage of ulcer reduction) and vitamin D levels, including variations in 25(OH)D levels between patients with diabetes and DFU, the odds ratio of DFU occurrence in diabetic patients, and the size of ulcer areas. Only meta-analysis papers published in English that explored the association between vitamin D and DFU outcomes and reported effect sizes (ES) along with their corresponding confidence intervals (CI) were considered for inclusion. Original papers, editorials, letters to the editor, and observational studies were excluded from consideration.

### 2.3 Study selection and data extraction

Meta-analyses were independently screened, and data from the identified papers were extracted by two reviewers (NG, NR) based on pre-established criteria. The initial screening of titles and abstracts determined eligibility, followed by the retrieval and assessment of full texts for potential inclusion. These stages were conducted independently by the authors, and any discrepancies regarding inclusion or exclusion were resolved by the final decision of the corresponding author. The extracted data included the author’s name, year of publication, health status of participants, study sample size, gender, mean age, outcomes measured, effect size (ES) and confidence intervals (CI) for DFU-related outcomes, and the quality of the included studies.

### 2.4 Methodological quality assessment

Two independent evaluators (MJ, PJ) appraised the methodological integrity of the included meta-analysis using the Assessing the Methodological Quality of Systematic Reviews 2 (AMSTAR2) checklist (16) and disagreements were resolved by the third author (QW). The AMSTAR2 questionnaire comprises 16 items that are answered with “Yes,” “Partial Yes,” “No,” or “Not a Meta-analysis.” The AMSTAR2 checklist is divided into four categories: “Critically low quality,” “Low quality,” “Moderate quality,” and “High quality.” **High quality:** A systematic review that meets all or nearly all criteria is rated as high quality. It has minor or no limitations, suggesting that the results are reliable. **Moderate quality:** A review that has more than one weakness but no critical flaws. It generally adheres to the most important methodological principles but has some shortcomings that are not likely to significantly change the results or conclusions. **Low quality:** A review that has one or more critical flaws according to the AMSTAR 2 criteria, with at least one of these flaws likely impacting the validity of the conclusions about at least one major outcome. The results should be interpreted with caution. **Critically low quality:** A review with more than one critical flaw that appears in multiple key areas, making the results of the review unreliable for drawing any conclusions.

## 3 Result

### 3.1 Study selection

Initially, we found 29 papers. Once we got rid of the duplicates, we had 20 studies left to look at more closely. We checked out the titles and summaries of these papers and picked out 13 articles that seemed worth a deeper look. In the end, after going through the full content of these 13 articles, we chose 7 of them based on specific criteria we had set from the start (14, 17–22).

### 3.2 Study characteristics

This umbrella review synthesized findings from seven meta-analyses Conducted between 2019 and 2023 that included twenty unique RCTs and explored the correlation between vitamin D levels and diabetic DFU. These analyses encompassed subjects of both sexes, aged 55 to 60. Among them, one meta-analyses exclusively reviewed randomized controlled trials (RCTs) (18), three combined observational studies with RCTs (19–21), two focused solely on observational studies (14, 17), and one was dedicated to nested case–control studies (22). The majority, six of the analyses, juxtaposed the 25(OH)D levels in DFU patients against individuals with diabetes but without ulcers (14, 17, 19–22). Meanwhile, a singular study contrasted DFU patients’ vitamin D levels with those of healthy controls (19). Furthermore, four meta-analyses quantified the risk (odds ratio) of DFU in the context of severe vitamin D deficiency (VDD) (<12 ng/mL) (14, 20–22), and one delineated the odds ratio for DFU at a moderate vitamin D deficiency range (12–20 ng/mL) (21). Another meta-analysis investigated the effect of vitamin D supplementation on ulcer areas (18). Table 1 provides a comprehensive report on the detailed information of the included meta-analyses.

**Table 1:** Characteristic of included studies.

Study, country, year	Included studies design	Participants	Study number	Gender	Mean age	Outcome	Quality of included studies
Kaissar Yammine et al. Lebanon 2019 (18)	Nested case–control	Diabetes vs. DFU	9	F/M	55.2	25(OH)D levels difference: WMD −0.93 ng/mL; 95% CI: −1.68, −0.17	7 studies: high quality, 2 study: low quality 5studies: high quality
	Nested case–control	Diabetes	5	F/M	55.2	OR 3.6; 95% CI, 2.94, 4.41 (reference value: sever VDD)	
Jiezhi Dai et al. China 2019 (17)	Observational	Diabetes vs. DFU	7	F/M	56	25(OH)D levels difference: MD −13.47 ng/mL; 95% CI: −16.84, −10.10	6 studies: high quality, 1 study: low quality 3 studies: high quality, 1 study: low quality
	Observational	Diabetes	4	F/M	53.8	OR 3.22; 95% CI, 2.42, 4.28 (reference value: sever VDD)	
Juan Lin et al. China 2022 (23)	RCT & Observational	Diabetes vs. DFU	11	F/M	-	25(OH)D levels difference: MD −6.48 ng/mL; 95% CI: −10.84, −2.11	14 studies: high quality, 6 study: low quality 5 studies: moderate quality
	Observational	Diabetes	7	F/M	-	OR 2.53; 95% CI, 1.65, 3.89 (reference value: sever VDD)	
	Observational	Diabetes	7	F/M	-	OR 1.82; 95% CI, 1.32, 2.52 (reference value: VDD)	
Xin Li et al. China 2023 (19)	RCT & Observational	Diabetes vs. DFU	15	F/M	-	25(OH)D levels difference: MD −7.14 ng/mL; 95% CI, −8.83, −5.44	12 studies: high quality, 9 study: low quality 4 studies: moderate quality
	Observational	Diabetes	10	F/M	-	OR 2.27; 95% CI, 1.63, 3.16 (reference value: sever VDD)	
Shilia Jacob Kurian et al. India 2023 (12)	RCT & Observational	Diabetes vs. DFU	13	F/M	-	25(OH)D levels difference: MD −5.41 ng/mL; 95% CI: −8.06, −2.76	11 studies: high quality, 2 studies: moderate quality 4studies: high quality, 1 study: moderate quality
	RCT & Observational	Diabetes vs. Healthy	5	F/M	-	MD −10.82 ng/mL; 95% CI: −20.47, −1.16	
Muhammad Iqhrammullah et al. Indonesia 2024 (20)	Observational	Diabetes vs. DFU	19	F/M	59	25(OH)D levels difference: SMD −1.28; 95% CI: −2.08, −0.47	16 studies: high quality, 3 studies: low quality
Edwin Kinesya et al. Indonesia 2023 (19)	RCT	DFU vs. DFU (25,000 & 60,000 IU/w vs. placebo) (12 weeks)	2	F/M	60	Ulcer area: MD: −2.70 cm; 95% CI: −2.90, −2.50	2 studies: low quality

DFU: diabetic foot ulcer; VDD: vitamin D deficiency; NOS: New Castle Ottawa; WMD: weight mean difference; OR: odds ratio.

### 3.3 Quality of the meta-analyses

According to AMSTAR2, two papers were assessed as critically low quality (18, 22) and five were low quality (14, 17, 19–21). Question number 7 was the most repeated item that did not meet expected standards and number 3 was the most reported item that met expected standards (Table 2).

**Table 2:** Quality assessment of included studies.

	Q1	Q2	Q3	Q4	Q5	Q6	Q7	Q8	Q9	Q10	Q11	Q12	Q13	Q14	Q15	Q16	Overall
Kaissar Yammine et al.	Yes	Partial Yes	Yes	Partial Yes	Yes	Yes	No	Yes	Yes	No	No	No	Yes	Yes	Yes	Yes	Critically low
Jiezhi Dai et al.	Yes	Partial Yes	Yes	Partial Yes	Yes	Yes	No	Partial Yes	Yes	No	Yes	Yes	Yes	Yes	Yes	Yes	Low
Juan Lin et al.	Yes	Partial Yes	Yes	Partial Yes	No	No	No	No	Yes	No	Yes	Yes	Yes	Yes	Yes	Yes	Low
Xin Li et al.	Yes	Partial Yes	Yes	Partial Yes	No	No	No	No	Yes	No	Yes	Yes	Yes	No	Yes	Yes	Low
Shilia Jacob Kurian et al.	Yes	Yes	Yes	Partial Yes	Yes	Yes	No	Partial Yes	Yes	No	Yes	Yes	Yes	No	Yes	Yes	Low
Edwin Kinesya et al.	Yes	Partial Yes	Yes	Partial Yes	No	Yes	No	Yes	Yes	No	No	Yes	Yes	No	No	Yes	Critically low
Muhammad Iqhrammullah et al.	Yes	Yes	Yes	Partial Yes	Yes	No	No	Yes	Yes	No	Yes	Yes	Yes	Yes	Yes	Yes	Low

1- Did the research questions and inclusion criteria for the review include the components of PICO? 2- Did the report of the review contain an explicit statement that the review methods were established prior to the conduct of the review, and did the report justify any significant deviations from the protocol? 3- Did the review authors explain their selection of the study designs for inclusion in the review? 4- Did the review authors use a comprehensive literature search strategy? 5- Did the review authors perform study selection in duplicate? 6- Did the review authors perform data extraction in duplicate? 7- Did the review authors provide a list of excluded studies and justify the exclusions? 8- Did the review authors describe the included studies in adequate detail? 9- Did the review authors use a satisfactory technique for assessing the risk of bias (RoB) in individual studies that were included in the review? 10- Did the review authors report on the sources of funding for the studies included in the review? 11- If meta-analysis was performed, did the review authors use appropriate methods for the statistical combination of results? 12- If a meta-analysis was performed, did the review authors assess the potential impact of RoB in individual studies on the results of the meta-analysis or other evidence synthesis? 13- Did the review authors account for RoB in individual studies when interpreting/ discussing the review results? 14- Did the review authors provide a satisfactory explanation for and discussion of any heterogeneity observed in the review results? 15- If they performed quantitative synthesis, did the review authors conduct an adequate investigation of publication bias (small-study bias) and discuss its likely impact on the review results? 16- Did the review authors report any potential sources of conflict of interest, including any funding they received for conducting the review?

### 3.4 Differences in 25(OH)D levels between patients with diabetes and DFU

Six investigations revealed a marked decrease in vitamin D levels among patients with diabetic foot ulcers (DFU) in comparison to individuals with diabetes but no ulcers, indicating significantly reduced 25(OH)D concentrations in those with DFU (14, 17, 19–22). Specifically, DFU patients exhibited a range decrease from −7.14 (5.44, 8.83) to −0.93 (95% CI, 0.17, 1.68) ng/ml in 25(OH)D levels relative to those suffering from diabetes alone. Furthermore, an analysis contrasting the vitamin D levels of DFU patients with healthy subjects found a similar trend (19) where DFU patients had 25(OH)D levels that were, −10.82 ng/mL (95% CI, −20.47 to −1.16) lower than those observed in the healthy control group.

### 3.5 The odds ratio of DFU for diabetes patients with vitamin D deficiency

Four meta-analyses found that individuals with diabetes who suffer from severe VDD are at a higher risk of developing DFU (14, 20–22). Individuals with severe VDD were, on range from OR: 1.82; (95% CI, 1.32 to 2.52) to OR: 3.6; (95% CI, 2.94, 4.41), at risk of DFU compared to those with diabetes alone. In addition, the study reporting the OR for DFU in those with VDD obtained a similar outcome (21). In this study, individuals with VDD had a 1.82 times higher risk of DFU compared to those with diabetes.

### 3.6 Effect of vitamin D on ulcer size in patients with DFU

In an investigation by Kinesya et al., the impact of vitamin D supplementation was assessed on an ulcer area. The result showed a significant diminution in the area of the ulcer MD: −2.70 cm (95% CI: −2.90 to −2.50) (18).

## 4 Discussion

We conducted a comprehensive search on the relationship between vitamin D levels and DFU observed at systematic reviews. In this study we observed that the 25-OH-vitamin D deficiency was higher in the diabetic foot group in most of the reviews evaluated. Despite the low quality of evidence, this effect was consistent when comparing DFU patients and non-DFU. The studies reviewed consistently point to the role of Vitamin D in enhancing the body’s immune response and reducing inflammation, which is critical in diabetic foot ulcer (DFU) management. Vitamin D helps modulate the activity of immune cells and the production of cytokines, which can influence healing processes. Patients with diabetes tend to develop chronic inflammation due to a decreased balance between pro- and anti-inflammatory factors. In addition, there is often an imbalance of oxidative stress induced by diabetes, which contributes to initiating inflammatory response cascades (23). This pathological inflammation contributes to the development of neuropathy and ischemia (24), through tissue dysfunction, decreased vasodilation, disturbed neovascularization and the formation of atherosclerotic plaques, which also act as a positive feedback loop (25).

Research indicates that vitamin D acts as an anti-inflammatory agent by promoting the development of monocytes into macrophages, while also diminishing their capacity to present antigens to T cells (26). Additionally, it hinders the maturation of dendritic cells (DC), leading to the creation of tolerogenic DCs that lack surface MHC molecules, rendering them incapable of antigen presentation. This impairment in antigen presentation by antigen-presenting cells leads to T cell anergy, which inhibits or reduces B cells’ proliferation, their evolution into plasma cells, the creation of memory B cells, and the synthesis of immunoglobulins, including autoantibodies. Moreover, calcitriol facilitates the transformation of CD4+ T cells into Th2 and regulatory T cells, while it diminishes the generation of Th1 and Th17 cells, effectively decreasing the Th1/Th2 ratio (27). Vitamin D also influences cytokine production, stimulating immune cells to release anti-inflammatory cytokines like IL-4, IL-10, and TGF-*β*, and simultaneously decreasing the output of pro-inflammatory cytokines such as IL-1β, IL-6, IL-12, IL-17, IL-22, TNF-*α*, and IFN-*γ* (28). Furthermore, reduced levels of circulating 25(OH)D may lead to heightened levels of inflammatory cytokines in patients with diabetic foot ulcers, thereby hindering the healing process (29). Vitamin D supplementation for six months increased the level of vitamin D and consequently suppressed serum levels of pro-inflammatory cytokines, namely IL-18, TNF-a and IFN-g. Taken together, vitamin D deficiency may contribute to the development of DFU, along with worsening chronic inflammation in diabetic patients (30). According to the last update of The International Working Group on the Diabetic Foot (31), pharmacological doses of vitamins should not be prescribed. Instead, only diabetic patients with low levels of vitamin D who are experiencing vitamin D deficiencies should be recommended supplements with normal doses. This recommendation aligns with the findings of our study, indicating that normal doses are more effective than high doses for addressing deficiencies in diabetic individuals without causing overcompensation. While this guideline does not advocate for vitamin D supplementation to directly reduce wound size, it does recommend it for mitigating neurodegenerative complications in patients with DFU. Kurian and collaborators (12) suggest that vitamin D supplementation to have a protective role in the immune and vascular system, improve glycemic outcomes, and wound healing. Therefore, vitamin D supplementation could be a preferred adjuvant in the management of DFU. Although meta-analyses (8, 18) are not completely certain about wound healing, it seems that supplementation with usual doses is useful in compensating insufficient levels of vitamin D. This summary identifies both limitations and strengths within its methodology. Key limitations stem from inadequate patient and outcome assessor blinding, along with a significant number of participant dropout’s post-randomization. Other issues include the absence of prior meta-analyses pre-registration, protocol alterations in some cases, and a lack of funding source disclosure in most instances. Given the findings, there’s a strong case for screening for Vitamin D deficiency in patients with diabetes, particularly those at risk for or currently managing DFUs. Suggesting guidelines for Vitamin D supplementation could be a practical step, helping to standardize care and potentially improve outcomes in DFU management.

One key methodological challenge in this study stems from the potential overlap among the original papers included in the meta-analyses, leading to insufficient analysis. The use of an umbrella meta-analysis introduces the risk of duplicating the findings from the incorporated studies. Despite this, the review demonstrates notable strengths, such as its rigorous methodological approach, extensive database search, and comprehensive analysis. In addition to comparing DFU in patients with diabetes, the review also includes stratified analyses that assess the impact of vitamin D on DFU in comparison to healthy individuals. The meta-analyses included in this review encompass a range of study designs—randomized controlled trials (RCTs), observational studies, and case–control studies—which introduce variability in the robustness of the findings. RCTs, considered the gold standard for clinical evidence, offer stronger causality but are often limited by small sample sizes, short follow-up periods, and selection bias, as seen in studies like Kinesya et al. (18). These limitations can undermine the external validity of their findings. Conversely, observational studies, such as those by Dai et al. (14), Iqhrammullah et al. (17), and Iqhrammullah et al. (17), provide data from larger populations and real-world settings but are more prone to confounding factors, making it difficult to establish definitive causal relationships between vitamin D deficiency and diabetic foot ulcers (DFU). While efficient for studying rare outcomes like DFUs, case–control studies face challenges such as recall bias and selection bias, impacting the accuracy of their findings. The inclusion of these diverse study designs introduces heterogeneity, which must be taken into account when interpreting the results. Studies that combine RCTs and observational data, like those by Lin et al. (21) and Li et al. (20), provide a more comprehensive view but at the cost of increased variability. While observational studies offer broader insights, they lack the rigor of RCTs. This diversity in study design necessitates a cautious interpretation of the findings, as the robustness of the conclusions may be influenced by the inherent limitations of each study type. The results suggest the need for more high-quality, long-term RCTs to confirm the role of vitamin D in managing DFUs and provide clearer guidance for clinical practice.

### 4.1 Implications for clinical practice

The findings from the umbrella review underscore the importance of incorporating vitamin D status into the management of DFUs. Healthcare providers should consider regular monitoring of serum vitamin D levels in diabetic patients, especially those at risk of developing DFUs. The meta-analyses consistently show that patients with DFUs tend to have significantly lower vitamin D levels compared to non-ulcerated diabetic patients, and severe vitamin D deficiency is associated with an increased risk of DFUs (14, 20, 21). Given the potential role of vitamin D in enhancing wound healing and modulating immune responses, healthcare providers may consider vitamin D supplementation as part of a comprehensive treatment strategy for DFUs. However, further clinical trials are needed to establish optimal dosing and assess the direct impact of supplementation on DFU outcomes.

## 5 Conclusion

In summary, the available evidence suggests a potential link between lower levels of vitamin D and diabetic foot ulcers (DFU), as indicated by various papers. Additionally, there seems to be an association between severe vitamin D deficiency and increased risk of DFU development in individuals with diabetes. Moreover, some meta-analyses suggest that vitamin D supplementation might have a role in reducing ulcer size in patients with DFU. However, due to potential biases in the included meta-analyses, the relationship between vitamin D levels and DFU should be interpreted cautiously, and further research is warranted to confirm these findings.

## Data Availability

The original contributions presented in the study are included in the article/Supplementary material, further inquiries can be directed to the corresponding author.
