# Supplementary figures and images for: The role of vitamin D in diabetic foot ulcer; an umbrella review of meta-analyses
Source: Front Nutr. 2024 Oct 9;11:1454779. doi: 10.3389/fnut.2024.1454779 (PMC11497990; doi:10.3389/fnut.2024.1454779)

## Quality assessment of included studies

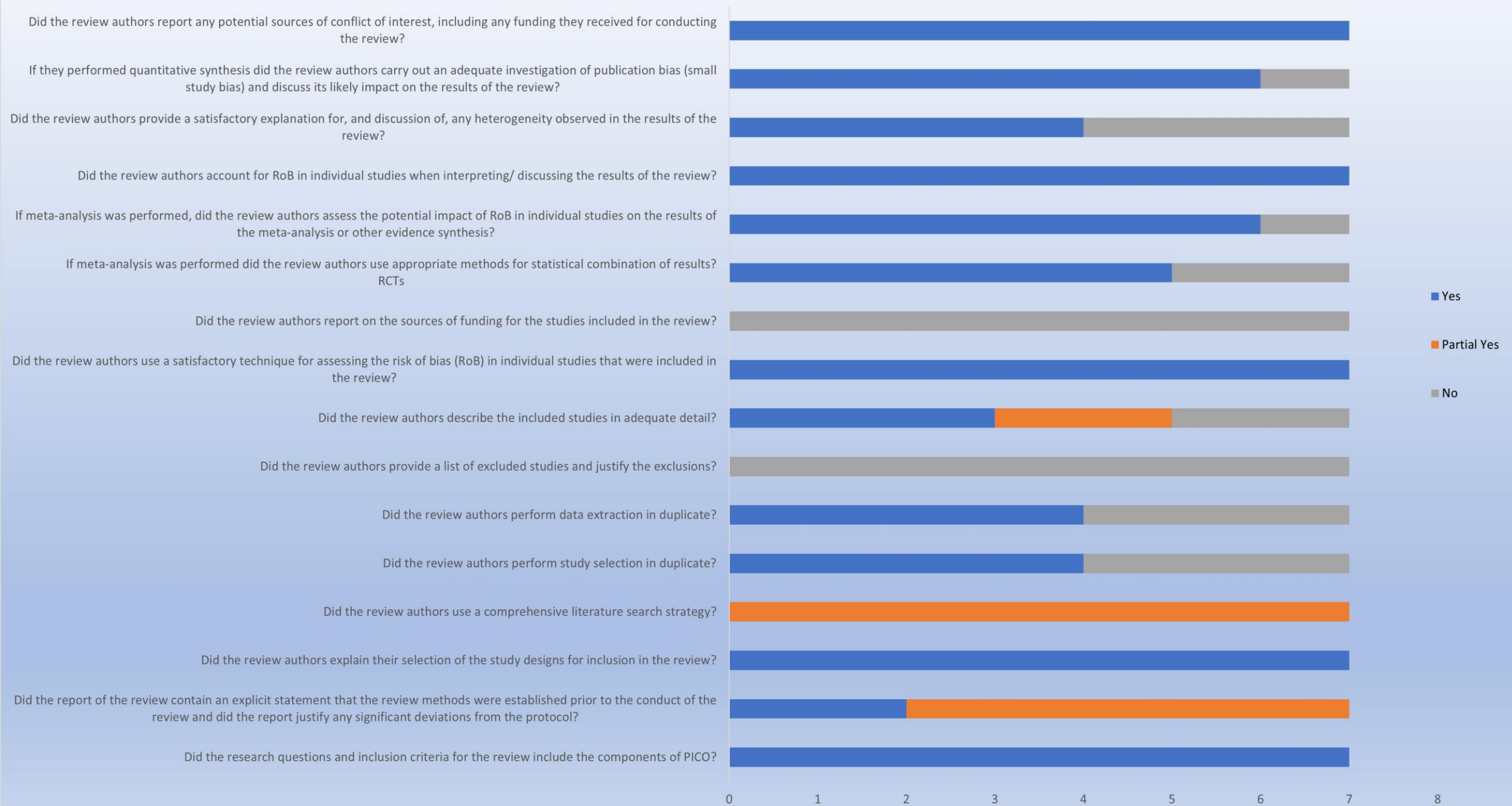

Supplement: Supplementary file 1 [file Data_Sheet_1.pdf]
